# Rolling Bearing Fault Diagnosis Based on WGWOA-VMD-SVM

**DOI:** 10.3390/s22166281

**Published:** 2022-08-21

**Authors:** Junbo Zhou, Maohua Xiao, Yue Niu, Guojun Ji

**Affiliations:** 1 College of Engineering, Nanjing Agricultural University, Nanjing 210032, China; 2 Essen Agricultural Machinery Changzhou Co., Ltd., Changzhou 213000, China

**Keywords:** fault diagnosis, VMD, SVM, rolling bearing, WGWOA

## Abstract

A rolling bearing fault diagnosis method based on whale gray wolf optimization algorithm-variational mode decomposition-support vector machine (WGWOA-VMD-SVM) was proposed to solve the unclear fault characterization of rolling bearing vibration signal due to its nonlinear and nonstationary characteristics. A whale gray wolf optimization algorithm (WGWOA) was proposed by combining whale optimization algorithm (WOA) and gray wolf optimization (GWO), and the rolling bearing signal was decomposed by using variational mode decomposition (VMD). Each eigenvalue was extracted as eigenvector after VMD, and the training and test sets of the fault diagnosis model were divided accordingly. The support vector machine (SVM) was used as the fault diagnosis model and optimized by using WGWOA. The validity of this method was verified by two cases of Case Western Reserve University bearing data set and laboratory test. The test results show that in the bearing data set of Case Western Reserve University, compared with the existing VMD-SVM method, the fault diagnosis accuracy rate of the WGWOA-VMD-SVM method in five repeated tests reaches 100.00%, which preliminarily verifies the feasibility of this algorithm. In the laboratory test case, the diagnostic effect of the proposed fault diagnosis method is compared with backpropagation neural network, SVM, VMD-SVM, WOA-VMD-SVM, GWO-VMD-SVM, and WGWOA-VMD-SVM. Test results show that the accuracy rate of WGWOA-VMD-SVM fault diagnosis is the highest, the accuracy rate of a single test reaches 100.00%, and the accuracy rate of five repeated tests reaches 99.75%, which is the highest compared with the above six methods. WGWOA plays a good optimization role in optimizing VMD and SVM. The signal decomposed by VMD is optimized by using the WGWOA algorithm without mode overlap. WGWOA has the better convergence performance than WOA and GWO, which further verifies its superiority among the compared methods. The research results can provide an effective improvement method for the existing rolling bearing fault diagnosis technology.

## 1. Introduction

Rolling bearing is the basic component of mechanical equipment and is used by most rotating machinery; it plays an important role in various fields of production [1,2]. Rolling bearing failure is likely to occur because it often operates under heavy load [3,4]. Statistics show that about 30% of mechanical faults in rotating machinery equipment using rolling bearings are related to bearing damage [5]. Fault diagnosis using vibration signals generated during its working process can reduce the probability of mechanical equipment accidents and provide reliable decision support for equipment later maintenance plans, which has high practical importance [6,7,8].

Rolling bearing vibration signal is nonlinear and complex due to many factors, so extracting effective information from it is particularly important [9,10,11]. Traditional methods mainly include time-domain and frequency-domain analyses. The former extracts and analyzes the statistical indexes of signals. The latter converts the signal into a frequency domain by Fourier transform and uses the fault frequency in the spectrum to make further analysis. However, the statistical characteristics of nonstationary complex signals in time and frequency domains must be analyzed and processed because they all change with time [12]. Common time-frequency analysis methods mainly include wavelet transform, empirical mode decomposition (EMD), and local mean decomposition (LMD) [13,14,15]. EMD can adaptively decompose the signal into several eigenmode functions. Song et al. [16] proposed a novel bearing fault diagnosis based on EMD and improved Chebyshev distance and verified its accuracy and robustness by experiments. However, EMD is prone to problems, such as end-point effect and mode overlap [17,18]. Compared with EMD, LMD improves the number of iterations and the speed of operation, but it still cannot solve the problems of end-point effect. In 2014, Konstantin et al. [19] proposed a new variable scale processing method called variable mode decomposition (VMD) [20]. This method introduces a variational model and converts signal decomposition into an optimization problem of constrained model, which can avoid the end-point effect, restrain mode confusion, and has high decomposition efficiency. However, selecting the decomposition level and secondary penalty factor accurately is difficult in the application of VMD. Lin et al. [21] conducted gear fault diagnosis by using cuckoo search (CS) to optimize VMD and probabilistic neural network. The test results show that VMD can effectively avoid mode overlap, and the accuracy of this fault diagnosis method is 98.50%. However, the kurtosis index is selected as the fitness function of the CS algorithm, which results in unstable values of the decomposition layer.

With the development of machine learning and deep learning, the combination of intelligent learning algorithm and rolling bearing fault identification has become a hot research topic. The commonly used methods are artificial neural network (ANN), backpropagation neural network (BPNN), and support vector machine (SVM) [22,23,24,25]. As a classical algorithm in machine learning, SVM can solve the problems of ANN easily being fitted and BPNN easily falling into local optimum, so SVM is widely used in pattern recognition [26,27,28]. Van et al. [29] built a hybrid SVM model and applied it to bearing fault classification. They proved its superiority in terms of classification effect and training time by experiment. The selection of the structural parameters of SVM is difficult and directly affects its performance. Particle swarm optimization (PSO), whale optimization algorithm (WOA), gray wolf optimization (GWO), and other representative population algorithms are widely used in the optimization of SVM structural parameters [30,31] due to their advantages, such as good optimization performance and easy implementation. Garca et al. [32] proposed a PSO-SVM model to predict the remaining service life of aircraft engines. The test results show that the prediction accuracy is higher than that of the traditional PSO-SVM method. Dong et al. [33] presented a rolling bearing fault diagnosis model of GWO-SVM. The test results show that the GWO-SVM fault diagnosis model is more efficient than the SVM model. However, the PSO algorithm has the limitation of being trapped in local optimum [34], and the GWO algorithm has low optimization accuracy. Thus, these algorithms can still be improved. He et al. [35] developed an improved WOA algorithm called SWOA to optimize SVM and applied it to the prediction of soil moisture in maize. The test results show that the mean absolute error of the predicted results of this method is reduced from 0.87 to 0.67 compared with the optimized SVM of WOA, which proves the feasibility of the improved algorithm.

Although VMD improves in terms of the end-point effect, mode mixing, and other issues, selecting the decomposition layers and secondary penalty factors accurately is difficult. The SVM model is suitable for fault classification, but its performance largely depends on the constraints of core function parameters and penalty factors. At present, population optimization algorithm has certain parameter optimization capability, but its structure needs to be further improved to meet the actual needs.

We proposed a whale gray wolf optimization algorithm-VMD-SVM (WGWOA-VMD-SVM) for the fault diagnosis of rolling bearing. The vibration signal of rolling bearing is decomposed by VMD. A WGWOA algorithm based on WOA and GWO is presented. This algorithm is used to determine the best secondary penalty factor and decomposition layer number of VMD. The vibration signal of rolling bearing is decomposed into several components by using VMD optimized by WGWOA. The permutation entropies are extracted as feature vectors. SVM is used as the rolling bearing fault diagnosis model, and the WGWOA algorithm is used to solve the optimal penalty factor and core function parameters. The optimized SVM model is trained in accordance with the extracted feature vector, and the test sample output is obtained. The fault diagnosis methods in this paper were comprehensively evaluated in terms of time-frequency signal, optimized fitness curve, and fault diagnosis accuracy to verify the feasibility and practicability of the proposed algorithm by two test cases.

## 2. Theoretical Basis

### 2.1. VMD

The essence of VMD is to decompose the vibration signal into several amplitude frequency-modulated signals by frequency domain iteration. For a group of complex vibration signals, the optimal variational model constructed by VMD can be decomposed into a series of intrinsic mode functions (IMFs) through multiple iterative calculations. In other words, the modal function *u_k_*(*t*), *k* ∈ [1, *B*] is obtained with the minimum sum of *B* prediction bandwidths in the time series of the original signal.

Suppose a multifrequency signal *F* can be divided into *k* discrete time series *u_k_*(*t*) with limited bandwidth. Their corresponding central fundamental frequency band is *ω_k_*(*t*). The spectrum obtained from *u_k_*(*t*) has sparse characteristics. The specific steps of bandwidth calculation are as follows.

The analytical signal and unilateral spectrum of the decomposed eigenmode function signal of each order are calculated by using Hilbert transform, which can be expressed as

(1) $$\left[\delta \left(t\right)+\frac{j}{\pi \cdot t}\right]\cdot u_{k}\left(t\right)$$

Each modal signal is multiplied by an exponential term to make certain adjustments to its central frequency band:

(2) $$\left\{\left[\delta \left(t\right)+\frac{j}{\pi \cdot t}\right]\cdot u_{k}\left(t\right)\right\}\cdot e^{-j\cdot w_{k}\cdot t}$$

The gradient norm of the demodulated signal is calculated, and the bandwidth of each modal signal is estimated, which can be expressed as

(3) $${\left\|\partial_{t}\cdot \left\{\left[\delta \left(t\right)+\frac{j}{\pi \cdot t}\right]\cdot u_{k}\left(t\right)\right\}\cdot e^{-j\cdot w_{k}\cdot t}\right\|}_{2}^{2}$$

The center frequency and bandwidth obtained by the above equation are conditionally limited, that is, it should meet the requirements of minimizing the sum of the signal bandwidths of each IMF. Therefore, a constrained variational model should be developed.

(4) $$\left\{ \begin{array}{l} {\left\|\partial_{t}\cdot \left\{\left[\delta \left(t\right)+\frac{j}{\pi \cdot t}\right]\cdot u_{k}\left(t\right)\right\}\cdot e^{-j\cdot w_{k}\cdot t}\right\|}_{2}^{2}\\ s.t\sum_{k=1}^{K}u_{k}=f \end{array}\right.$$

where *ω_k_* is the frequency center of each IMF; *u_k_* is the *k*th IMF; *f* is the original signal.

The quadratic penalty factor method and the Lagrange function multiplier method are introduced to transform the above equation into an unconstrained variational problem and to obtain its optimal solution. The augmented Lagrange function is

(5) $$L(u_{k},\omega_{k},\lambda )=\alpha \cdot {\sum_{k=1}^{K}\left\|\partial_{t}\cdot \left\{\left[\partial (t)+\frac{j}{\pi \cdot t}\right]\cdot u_{k}(t)\right\}\cdot e^{-j\cdot \omega_{k}\cdot t}\right\|}_{2}^{2}+{\left\|f(t)-\sum_{k=1}^{K}u_{k}(t)\right\|}_{2}^{2}+\langle \lambda (t),f(t)-\sum_{k=1}^{K}u_{k}(t)\rangle$$

The alternating direction method of multipliers is introduced to search the saddle point of the variational problem. The center frequency and bandwidth of each IMF signal can be updated:

(6) $$\omega_{k}^{n+1}=\frac{{\int_{0}^{\infty }\omega \cdot \left|{\hat{u}}_{k}(\omega )\right|}^{2}d\omega }{\int_{0}^{\infty }{\left|{\hat{u}}_{k}(\omega )\right|}^{2}d\omega }$$

(7) $${\hat{u}}_{k}^{n+1}(\omega )=\frac{\hat{f}(\omega )-\sum_{i\neq k}{\hat{u}}_{i}(\omega )+\frac{\hat{\lambda }(\omega )}{2}}{1+2\cdot \alpha \cdot {\left(\omega -\omega_{k}\right)}^{2}}$$

where ${\hat{u}}_{k}^{n+1}(\omega )$ is the filtering result of residual quantity $\hat{f}(\omega )-\sum_{i\neq k}{\hat{u}}_{i}(\omega )$; $\omega_{k}^{n+1}$ is the power spectrum center of gravity of the current mode; and the real part can be obtained by inverse fast Fourier transformation of ${\hat{u}}_{k}(t)$.

The decomposition steps of VMD are as follows:

Step 1: Initialize parameter *u_k_*, *ω_k_*, *λ*, *α*, and *N*;

Step 2: *N* = *N* + 1, and the VMD algorithm is used for iterative calculation;

Step 3: The value of *k* is continuously superimposed from 1 to *k*, *u_k_* and *ω_k_* are continuously updated by using Equations (5) and (6), respectively, and *k* is the total amount of IMF finally decomposed;

Step 4: Update *λ* in accordance with the following equation:

(8) $$\lambda^{n+1}=\lambda^{n}+\tau \cdot (f-\sum_{k}{\hat{u}}_{k}^{n+1})$$

Step 5: Give the judgment accuracy *ε* > 0, and repeat steps (3) and (4) until the termination conditions of the following equation are met:

(9) $$\sum_{k}\frac{{\left\|u_{k}^{n+1}-u_{k}^{n}\right\|}_{2}^{2}}{{\left\|u_{k}^{n}\right\|}_{2}^{2}}\;<\;\varepsilon$$

VMD can effectively avoid the phenomenon of modal aliasing and can perform effective signal analysis to extract differentiated eigenvalues due to its strong robustness. Therefore, the signal after VMD can effectively describe the characteristics of fault signals.

### 2.2. WGWOA

GWO is an algorithm proposed by Mirjalili et al. [36]. Its basic principle is to imitate the population system of gray wolves and divide them into *α*, *β*, *δ*, and *γ.* Gray wolf *γ* accepts gray wolves during the hunting of *α*, *β*, and *δ*. The process of the gray wolf algorithm can be divided into three stages: encirclement, pursuit, and attack [37,38], and the specific steps are as follows:

Step 1: Surround prey

In the GWO algorithm, each gray wolf individual realizes prey encirclement in accordance with the following equation:

(10) $$D=\left|C\cdot x_{p(t)}-x_{t}\right|$$

(11) $$x_{t+1}=x_{p(t)}-A\cdot D$$

where *D* is the Euclidean distance between the wolf individual and its prey; *X*_(*p*(*t*))_ is the location of the prey; *X_t_* is the individual position of wolf before the start of the enclosure process; *X_t_*_+1_ is the individual position of the gray wolf at the end of the enclosure process.

The calculation equations of variable coefficients *A* and *C* are as follows:

(12) $$A=2\cdot a\cdot r_{1}-a$$

(13) $$C=2\cdot r_{2}$$

where *a* is the contraction factor, which decreases linearly from 2 to 0; *r*_1_ and *r*_2_ are two different [0, 1] random numbers.

Step 2: Hunt prey

After surrounding the prey, gray wolves *α*, *β*, and *δ* are three potential solutions. All the individuals in the wolf pack are in the GWO algorithm. *α*, *β*, and *δ* are led by prey hunting, and each gray wolf individual follows the following equations for pursuing:

(14) $$D_{q}=\left|C_{l}\cdot x_{j}-x_{f(t)}\right|$$

(15) $$x_{l}=x_{q}-A_{l}\cdot D_{q}$$

(16) $$x_{f(t+1)}=\frac{\sum x_{l}}{3}$$

where *q* takes *α*, *β*, and *δ*; *l* taken 1, 2, and 3; *Dq* is the Euclidean distance between the *q* wolf and the gray wolf. *x_l_* is the distance from the individual gray wolf to the *q* wolf; *x_f_*_(*t*)_ is the individual position of the gray wolf before the start of the chase; *x_f_*_(*t*+1)_ is the individual position of the gray wolf after the end of catching; *A_l_* and *C_l_* of the coefficient of variation are determined by using Equations (12) and (13), respectively.

Step 3: Attack prey

When the prey is surrounded by a pack of wolves, the pack begins attacking the prey. When *a* decreases linearly from 2 to 0, the range of *A* is [−*a*, *a*], as shown in Equation (12). When |*A*| < 1, the gray wolf is attacking its prey. When |*A*| > 1, the gray wolf leaves the wolf pack to find the next prey and expand the entire wolf pack search capability.

The GWO algorithm is nongreedy in nature, so it has good global optimization ability and is not easy to fall into local optimum. However, the GWO algorithm only uses straight-line hunting to catch prey, which restricts its search range and accuracy, resulting in slow convergence speed and poor local optimization capability. Therefore, the manner of the gray wolf algorithm to chase prey needs to be improved.

WOA is an algorithm that simulates whale predation in nature. It is divided into three stages: surround prey, bubble attack, and search-and-prey [39]. During the bubble attack phase, each individual chases its prey in a shrink enclosure with a 50% probability, similar to the way in which the individual chases its prey in the GWO algorithm (Equation (15)) and spirals its prey with a 50% probability. The whale algorithm uses the following methods of bubble attack:

(17) $$D_{w}=\left|x_{wbest}-x_{w}\right|$$

(18) $$x_{w+1}=\left\{ \begin{array}{lll} x_{wbest}-A_{w}\cdot D_{w} & & r_{3}<0.5\\ x_{wbest}+D_{w}e^{b\cdot R}\cdot \cos\left(2\cdot \pi \cdot R\right) & & r_{3}\geq 0.5 \end{array}\right.$$

where *D_w_* is the Euclidean distance between the individual whale and the best individual whale; *x_wbest_* is the position of the best individual whale; *x_w_* is the individual position of the whale before bubble attack; *x_w_*_+1_ is the individual position of the whale after bubble attack; *b* is the logarithmic spiral shape constant; *R* is a random number between [−1, 1]; *r*_3_ is a random number between [0, 1]; the variable coefficient *A_w_* is determined in the same manner as Equation (12).

Inspired by the bubbling attack mode of the WOA algorithm, the WGWOA algorithm is proposed. The stages of enclosing and attacking prey in this algorithm are consistent with the GWO algorithm, and the manner of chasing prey is as follows:

(19) $$D_{q}=\left|C_{l}\cdot x_{j}-x_{t}\right|$$

(20) $$x_{l}=\left\{ \begin{array}{lll} x_{q}-A_{l}\cdot D_{q} & & r_{3}<0.5\\ x_{q}+D_{q}e^{b\cdot R}\cdot \cos\left(2\cdot \pi \cdot R\right) & & r_{3}\geq 0.5 \end{array}\right.$$

(21) $$x_{t+1}=\frac{\sum x_{l}}{3}$$

where *x_t_* is the position before the start of the individual chase in the WGWOA algorithm; *x_t_*_+1_ is the position before the start of the individual chase.

Equations (19)–(21) show that the WGWOA algorithm still follows the wolf-led strategy of the gray wolf algorithm, retains the nongreedy algorithm with strong global optimization ability, and introduces the bubble attack mode of the WOA algorithm, which improves the population diversity, local optimization ability, and convergence performance. Therefore, the WGWOA algorithm considers the global and local optimization performance of the algorithm.

### 2.3. VMD Optimized Based on the WGWOA Algorithm

In the VMD process, the quadratic penalty factor *σ* and the number of IMF components *K* have a great influence on its decomposition results. The values of *σ* and *K* depend on the empirical parameters in the literature, which to a large extent has a tentative problem, and their applicability is limited. If the two parameters are not selected well, the signal will not be well-decomposed, resulting in over decomposition or under decomposition, which affects the extraction and judgment of important information.

Therefore, the VMD algorithm should be improved so that the appropriate *σ* and *K* values can be selected to realize the correct decomposition of the vibration signal of the rolling bearing. In this paper, the WGWOA algorithm is used to optimize the parameters *σ* and *K* of the VMD algorithm, and adaptive selection is performed to determine the best combination of parameters [*σ*, *K*].

Permutation entropy is a dimensionless index used to characterize the complexity of signal sequence and has many advantages, such as low requirement for sequence length and strong robustness. Therefore, it is widely used in condition monitoring, fault diagnosis, and signal detection of mechanical equipment [40]. Therefore, the permutation entropy of each component of VMD is used as the fitness function in the optimization of WGWOA algorithm due to the characteristics of permutation entropy.

Assuming a signal of length *L*: {*y*(*i*), *i* = 1, 2,…, *L*}, the permutation entropies are calculated as follows:

Step 1: Spatial reconstruction

(22) $$\left[\begin{array}{c} x\left(1\right)x\left(1+\tau \right)x\left[1+\left(m-1\right)\cdot \tau \right]\\ x\left(2\right)x\left(2+\tau \right)x\left[2+\left(m-1\right)\cdot \tau \right]\\ x\left(z\right)x\left(x+\tau \right)x\left[z+\left(m-1\right)\cdot \tau \right]\\ \ldots \\ x\left(\kappa \right)x\left(\kappa +\tau \right)x\left[\kappa +\left(m-1\right)\cdot \tau \right] \end{array}\right],z=1,2,\ldots ,\kappa \left[\kappa =L-\left(m-1\right)\cdot \tau \right]$$

where *m* is the embedding dimension; *τ* is the delay time.

Step 2: Reconstruct the *z*th reconstructed component *x*(*z*), *x*(*z* + *τ*),…, *x*[*z* + (*m* − 1)·*τ*] in ascending order. The values of *z*_1_, *z*_2_,…, *z_m_* indicate the index of the column in which each element in the reconstructed component is located. A set of symbolic sequences can be obtained for each row of the reconstruction matrix of any time series *y*(*i*) reconstructed from the phase space.

(23) $$S\left(\xi \right)=\left(z_{1},z_{2},\ldots ,z_{m}\right),\xi =1,2,\ldots ,\theta \left(\theta \leq m!\right)$$

Step 3: *m*-dimensional phase space is mapped to *m*!, and *S*(*ξ*) is only one of the different sequences of symbols. If the occurrence probability of each sequence of symbols is recorded as *P*_1_, *P*_2_,…, *P_θ_*, then the permutation entropy is calculated as follows:

(24) $$PE=-\sum_{i=1}^{\theta }P_{z}\cdot \ln P_{z}$$

**Figure 1:**
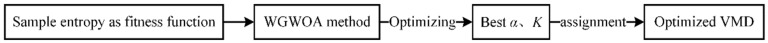
Flowchart of VMD optimization based on the WGWOA algorithm.

The process of VMD optimization based on the WGWOA algorithm is as Figure 1:

### 2.4. Fault Diagnosis Model Based on Optimized SVM

SVM is a machine learning method based on statistical learning theory. Its algorithm is characterized by maximizing the interval and it can find the optimal classification hyperplane [41] that separates different types of sample data and has the maximum classification interval. SVM can map input sample space to high-dimensional feature space by means of “core mapping,” overcome the problems of “dimension disaster” and “overfitting” in traditional machine learning model, and show great advantages in solving small sample, nonlinearity, and high-dimensional identification [42]. Therefore, SVM is used to construct a fault pattern recognition model. SVM needs to train the test data to realize fault identification, so the characteristic value of bearing vibration signal should be extracted to construct the data set. Based on the advantages of permutation entropy described above, the permutation entropy of each component after WGWOA-VMD decomposition is extracted to form a feature vector.

**Figure 2:**
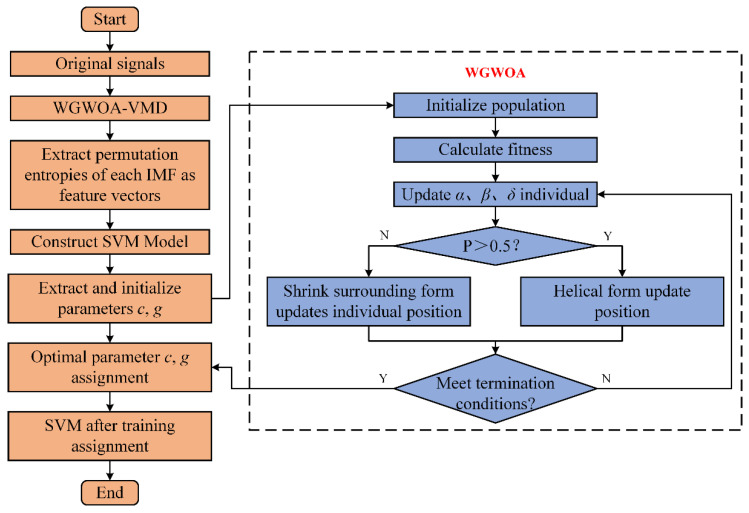
Flowchart of fault diagnosis based on the optimized SVM.

The penalty factor *c* and parameter *g* of radial basis core function have great influence on the performance of SVM during training. Therefore, the two parameters *c* and *g* of SVM are optimized by using the proposed WGWOA algorithm. A fault diagnosis model based on the optimized SVM was proposed. The flow chart of the algorithm is shown in Figure 2.

## 3. Fault Diagnosis of Rolling Bearing Based on WGWOA-VMD-SVM

**Figure 3:**
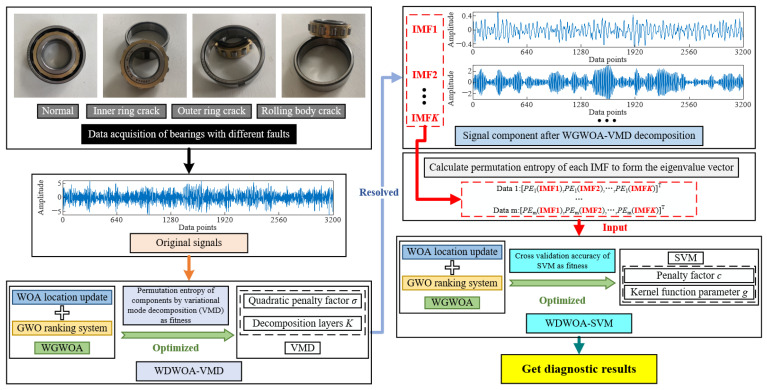
Fault diagnosis of rolling bearing based on WGWOA-VMD-SVM.

The rolling bearing fault signal is processed and recognized through signal processing, feature extraction, and pattern recognition. The general research route and basic theory are shown in Figure 3. In signal processing, the vibration signal is decomposed by VMD, the WGWOA algorithm is proposed to calculate the parameters in VMD, and *σ* is optimized with *K*. In feature extraction, the permutation entropy of each IMF decomposed by WGWOA-VMD is extracted to form the characteristic vector of vibration signal. In the aspect of fault pattern recognition, the characteristic vectors of each signal are inputted into the SVM model for fault diagnosis and classification, and the WGWOA algorithm is used to optimize the important parameters *c* and *g* of SVM.

The specific steps of rolling bearing fault diagnosis based on WGWOA-VMD-SVM are as follows:

Step 1: The fault states of normal rolling bearing, inner ring crack, outer ring crack, and rolling element crack are sampled many times;

Step 2: Taking the permutation entropy of each component VMD decomposed of signal samples as the fitness function, the WGWOA algorithm is used to decompose the input parameters of VMD in each fault condition by the number of levels *K* and the quadratic penalty factor *σ*. At the same time, input the vibration signal training samples, perform VMD, and obtain *K* eigenmode function components;

Step 3: The permutation entropy of *K* modes is extracted as the sample eigenvector, and the eigenvalue matrix is constructed;

Step 4: Taking the accuracy of SVM cross-validation as the fitness function, the proposed WGWOA algorithm is used to optimize the SVM parameters *c* and *g*;

Step 5: Input the test samples into the trained SVM, obtain the diagnostic results, and verify the training effect.

## 4. Experimental Research Based on Public Data Set

### 4.1. Test Data Acquisition

**Figure 4:**
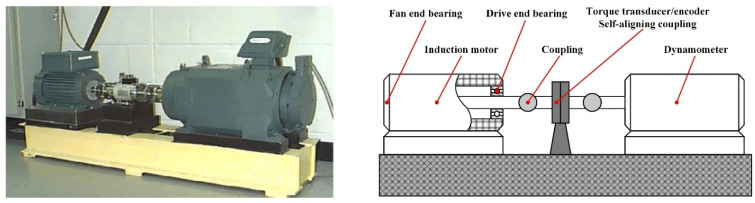
Rolling bearing fault simulation experimental device.

The bearing vibration signals collected by Case Western Reserve University were used as the experimental data, which was based on the test bench shown in Figure 4. In this experiment, the bearings at the motor drive end and the fan end were taken as the diagnostic objects, and the single point damage was introduced on the inner ring, outer ring, and roller of the test bearing by EDM to simulate three kinds of bearing faults. The damage size was 0.1778 mm, 0.3556 mm, and 0.5334 mm, respectively, and then the signals were collected by the acceleration sensor under different working conditions.

**Figure 5:**
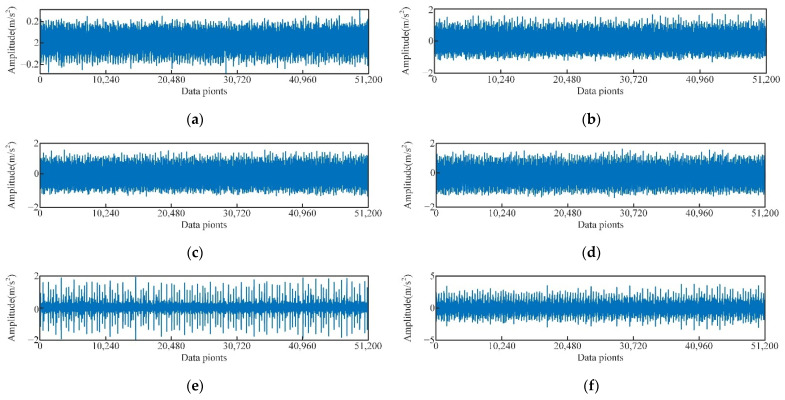

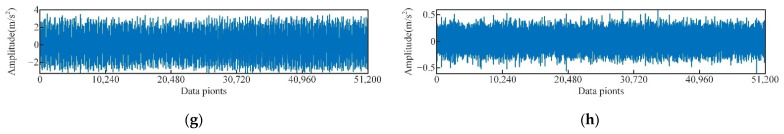
Time domain diagram of vibration signals of different types of rolling bearings, (**a**) 0HP load normal, (**b**) 0HP load inner ring fault diameter is 0.1778 mm, (**c**) 1HP load inner ring fault diameter is 0.1778 mm, (**d**) 2HP load inner ring fault diameter is 0.1778 mm, (**e**) 0HP load inner ring fault diameter is 0.3556 mm, (**f**) 0HP load inner ring fault diameter is 0.5334 mm, (**g**) 0HP load outer ring fault diameter is 0.1778 mm, (**h**) 0HP load rolling element fault diameter is 0.1778 mm.

In this test, the vibration signals of the rolling bearing at the driving end under normal condition, inner ring fault, outer ring fault, and roller fault with diameters of 0.1778 mm, 0.3556 mm, and 0.5334 mm, respectively, and loads of 0HP (speed 1796 r·min^−1^), 1HP (speed 1772 r·min^−1^), and 2HP (speed 1750 r·min^−1^) were analyzed. The sampling frequency was 12 kHz, the time domain diagram of some vibration signals of the test are shown in Figure 5.

**Figure 6:**
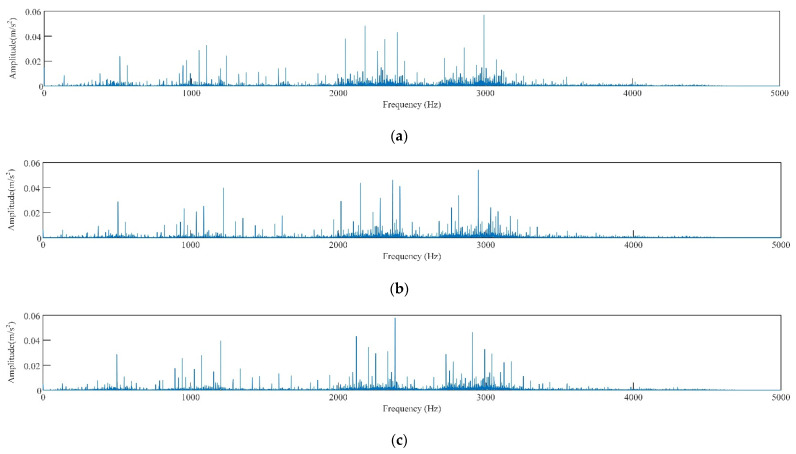
Frequency domain diagram of vibration signal of rolling bearing with inner ring fault under different loads, (**a**) 0HP, (**b**) 1HP, (**c**) 2HP.

**Figure 7:**
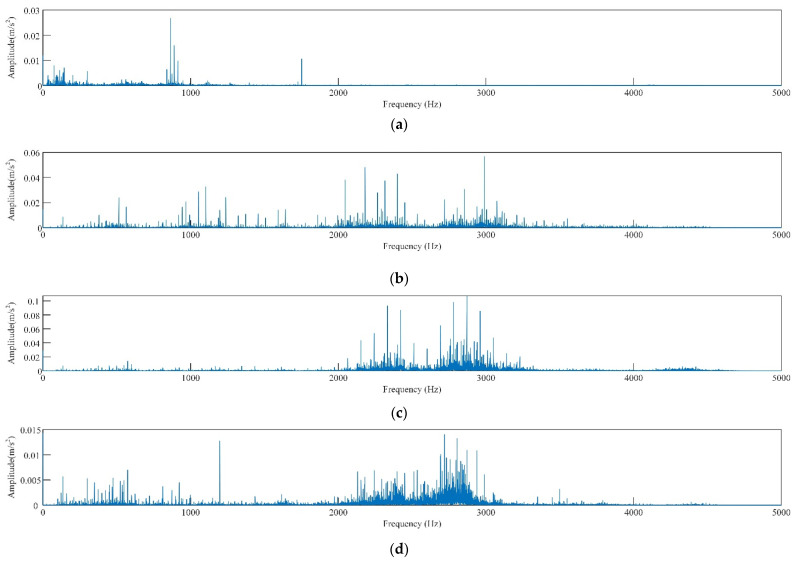
Frequency domain diagram of rolling bearing vibration signals of different fault types, (**a**) normal, (**b**) inner ring damaged, (**c**) outer ring damaged, (**d**) rolling body damaged.

As shown in Figure 5b–d, for the same fault type, the signal discrimination of rolling bearing under different loads is small. Comparing the spectrograms of corresponding signals shown in Figure 5b–d (Figure 6), the spectrograms of the three signals are also quite similar. This is because the rotational speeds of the experimental data under different loads are similar and the frequency of the characteristic pulse occurrence is very small, so the spectrum peaks of the fault characteristic frequency in the spectrogram are not significantly different either. As shown in Figure 5b,e, and f, there are significant differences in time domain waveforms of the vibration signal of the rolling bearing with different fault diameters [43]. Compared with normal bearings, the amplitude of faulty bearings and obvious periodic vibration impact are obvious, as shown in Figure 5a,b,g,h. In the spectrum diagram (Figure 7), the spectrum of normal bearing vibration signal is relatively single from Figure 7a, and the energy mainly concentrates in the low frequency band. Figure 7b,c shows that the energy of inner and outer ring fault vibration signal mainly concentrates in the middle frequency band, and the low frequency is reflected in the spectrum. It can be seen from the failure of the rolling body in Figure 7d. As shown in Figure 7d, when a rolling element fails, it is accompanied by more prominent energy in both low and medium frequency bands, The signal is also rather cluttered.

Although the vibration signals of different faults are different, these signals are only individual ideal signals. In fact, the waveforms of some states are very similar and difficult to distinguish. Therefore, it is necessary to further separate and extract the characteristics of vibration signals by mode decomposition of each signal.

### 4.2. Signal Processing and Feature Extraction

The test samples are set as follows: the vibration signals of 51,200 data points of each type are collected. Because the load of the rolling bearing will change under actual conditions, the vibration signals of three loads under the same fault type were randomly combined according to the load type to detect whether the method in this paper can identify the same fault under different loads. According to this, a total of 153,600 data points were obtained for each new combination signal. Vibration signals of 2048 data points were VMD decomposed, and the permutation entropy of each component was extracted as the characteristic vector. A total of 75 samples were obtained for each fault. Then, 45 samples were randomly selected as the training sample set, and the remaining 30 samples were used as the test sample set. Each test sample set is as shown in Table 1.

Taking each component permutation entropy of vibration signal VMD decomposed as the fitness function, the parameters *σ* and *K* in VMD were determined by WGWOA. The optimal parameters determined by the algorithm are shown in Table 2, the parameters of different vibration signals obtained by the algorithm are relatively centralized, the decomposition layers *K* are all four, and the secondary penalty factor *σ* fluctuates slightly around 2000. In order to guarantee the optimization effect and the universality of the method, when the fault type of vibration signal to be diagnosed is unknown, reasonable signal decomposition is carried out. In this paper, the best combination of parameters was determined by obtaining the average value of VMD optimal parameters of *σ* and *K* different vibration signals [1996.20, 4].

**Figure 8:**
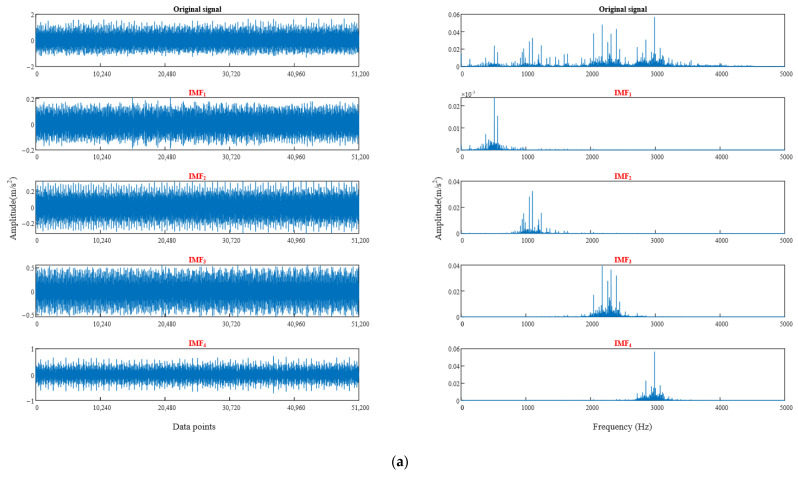

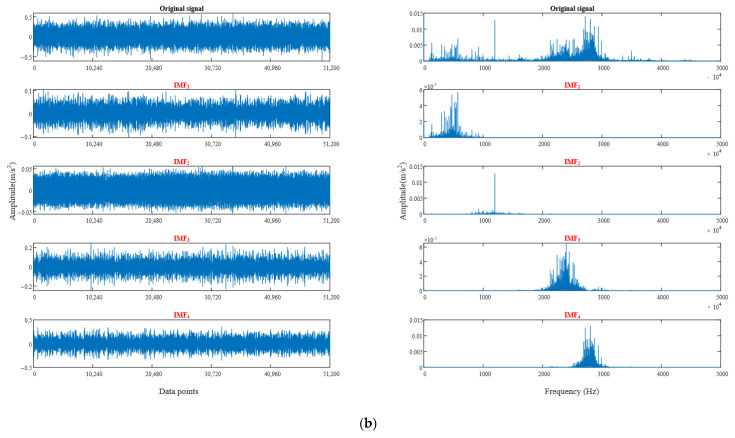
Time domain waveform and spectrum diagram of vibration signal decomposed by WGWOA-VMD, (**a**) inner ring fault, (**b**) rolling body fault.

In order to verify the rationality of selecting the best parameter combination, Figure 8 shows the time-domain waveform and spectrum of bearing vibration signal after using the best parameter VMD (only two signals are listed here due to the length of the article). From the spectrum diagram, the vibration signal of each component can accurately reflect the characteristics of the original signal after using the best parameter combination VMD, and there is no modal aliasing, which proves the feasibility of WGWOA-VMD.

### 4.3. Fault Diagnosis Results and Comparative Analysis

**Figure 9:**
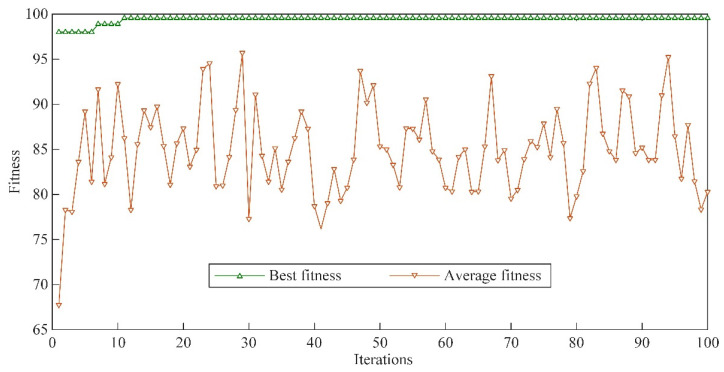
Fitness curve of SVM training process.

SVM is used as the fault diagnosis model, and the correct cross-validation is the fitness during SVM training. The WGWOA algorithm is used to optimize the parameters *c* and *g* of its SVM, and the final optimal *c*, *g* solution combination is [16.58, 3.83]. Figure 9 is the fitness curve of the SVM training process. The SVM is trained with training samples, and the test samples are input into the trained SVM to output the diagnosis results.

**Figure 10:**
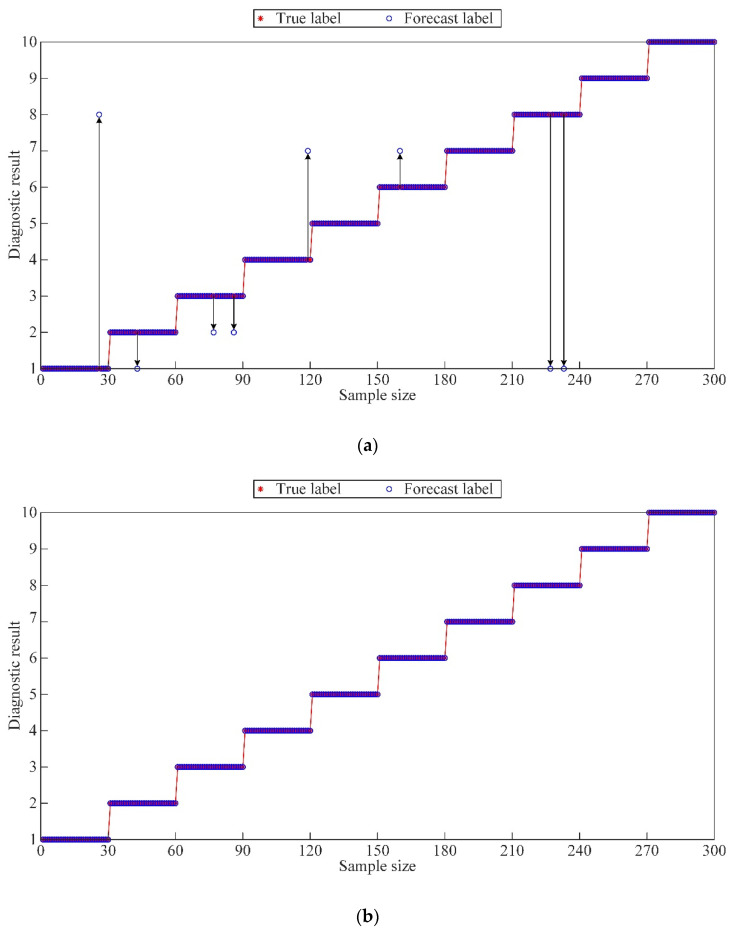
Fault diagnosis results of different methods, (**a**) VMD-SVM, (**b**) WGWOA-VMD-SVM.

In order to preliminarily verify the feasibility of the fault diagnosis method in this paper, the existing fault diagnosis method combining VMD and SVM was used for a comparative test. As shown in Figure 10, the fault diagnosis accuracy rate of WGWOA-VMD-SVM method in this paper reaches 100.00%, and the accuracy rate of VMD-SVM fault diagnosis method is 97.33%. This is because WGWOA-SVM adopts the WGWOA algorithm to optimize the parameters of VMD and enhance the effect of signal decomposition. At the same time, the WGWOA algorithm is used to optimize the parameters of SVM and improve the recognition ability of the SVM model. In order to avoid contingency, five repeated tests were carried out for the two fault diagnosis methods. The experimental results are shown in Table 3. The five fault diagnosis rates of WGWOA-VMD-SVM method are all 100.00%, indicating that the fault diagnosis method in this paper has strong stability.

## 5. Laboratory Test Research

### 5.1. Sources of Test Data

**Figure 11:**
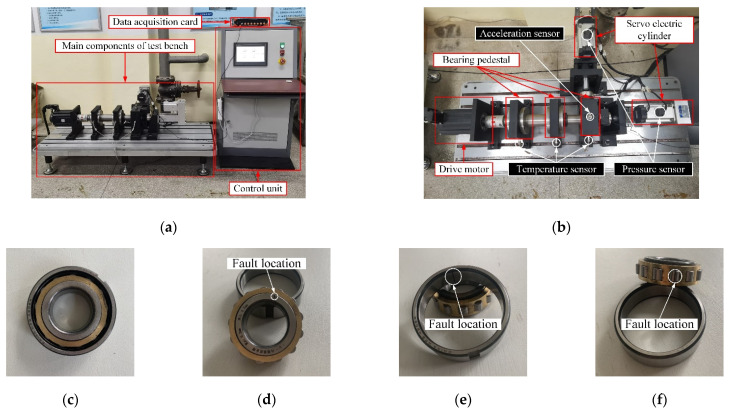
Test materials, (**a**) general layout of test stand, (**b**) schematic of the main structure of test stand, (**c**) normal bearings, (**d**) inner ring cracked bearings, (**e**) outer ring cracked bearings, (**f**) roller cracked bearings.

The bearing life cycle test platform of a mechanical transmission system independently developed by Nanjing Agricultural University was used for the test. As shown in Figure 11a,b, it is mainly composed of an integrated console, bearing pedestal, servo electric cylinder, motor, data acquisition card, acceleration sensor PCB35A26, temperature sensor, and pressure sensor. The motor speed and load are adjusted by the integrated console. During the test vibration signal collection, the motor drives the shaft to rotate, and the fault bearing is installed in the bearing seat of the shaft. The data acquisition card and the acceleration sensor are used to collect the bearing vibration data. The magnet at the bottom of the acceleration sensor is adsorbed in the radial direction of the bearing seat to be tested. After the test bench runs for 2 min, the running state is stable. The computer end acquisition software is used to start collecting the bearing vibration signal. App 2kN load to the motor through the load knob on the console and set the speed and sampling frequency to 1500 r·min^−1^ and 16 kHz, respectively. The bearing used in the test is a cylindrical roller bearing with the model of N205EM. The specific parameters are shown in Table 4. Regular cracks with a width of 0.2 mm and a depth of 0.5 mm were machined by EDM to simulate the fault bearing. The test bearing types include normal bearing, inner ring cracked bearing, outer ring cracked bearing, and rolling element cracked bearing (as shown in Figure 11c–f, and the quantity is one for each bearing).

### 5.2. Preprocessing of Test Data and Feature Extraction

#### 5.2.1. Data Preprocessing

The test data in this paper were set as follows: vibration signals of 80,000 data points in 5 s of each fault were collected, vibration signals of 1600 data points in 0.1 s were decomposed by VMD, permutation entropy of each IMF after VMD were extracted to form characteristic vectors, and 50 sets of data were obtained for each fault. Given that four types of faults are found in this test, 200 sets of data were set up to randomly divide the sample data sets of each fault condition in accordance with the proportion, avoiding phenomena, such as model fitting. Thirty groups (120 groups) of the bearing data of each state were used as training data for SVM, and the remaining 20 groups (80 groups) were used as test data for SVM.

**Figure 12:**
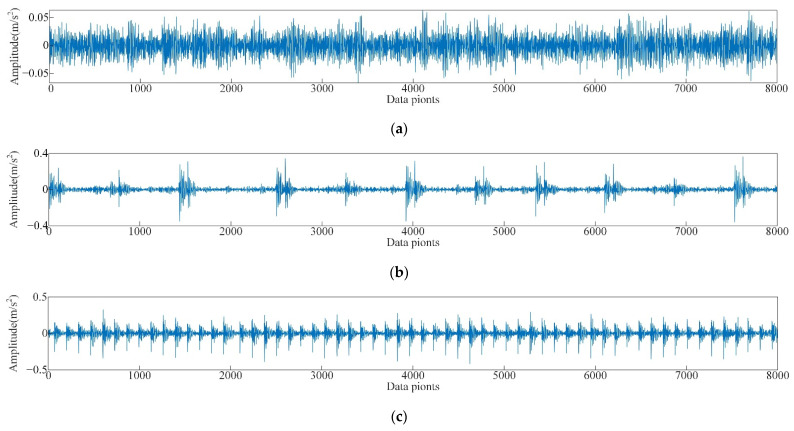

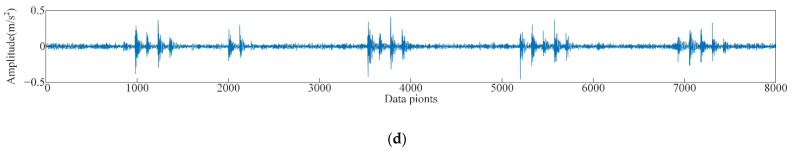
Time-domain waveform of the vibration signals of bearings with different faults, (**a**) normal bearings, (**b**) inner ring cracked bearings, (**c**) outer ring cracked bearings, (**d**) roller cracked bearings.

As shown in Figure 12, the vibration signal within 0.5 s (8000 data points) was collected for this experiment. As shown in the diagram, the vibration signal of normal bearing (Figure 12a) is relatively stable, with small amplitude and no large pulse. The vibration signals of faulty bearings (Figure 12b–d) differ from those of normal bearings. The time domain waveforms of fault bearing vibration signals have a larger amplitude and larger periodic vibration impact, various fault time domain diagrams are different, but it is not easy to determine the specific fault characteristics. Therefore, the characteristics of each vibration signal should be further separated and extracted by signal mode decomposition.

#### 5.2.2. Signal Decomposition and Feature Extraction Based on WGWOA-VMD

**Figure 13:**
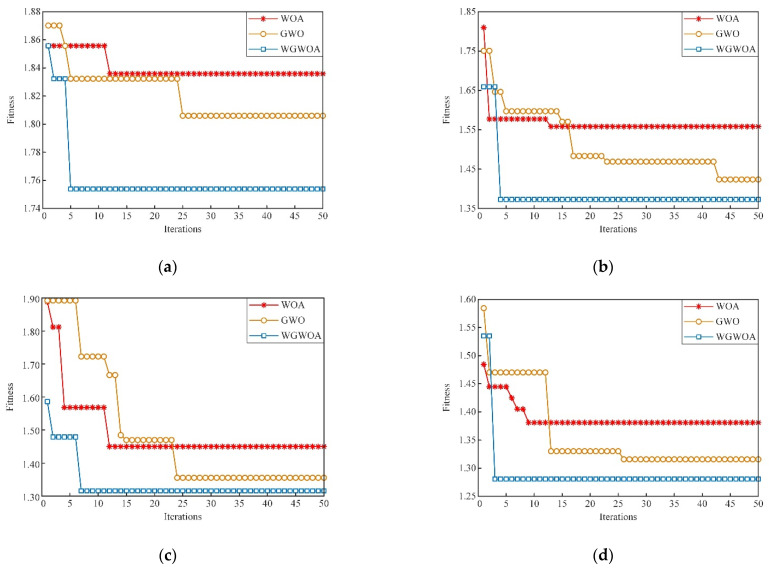
Different algorithms to optimize the fitness curve of VMD, (**a**) normal bearings, (**b**) inner ring cracked bearings, (**c**) outer ring cracked bearings, (**d**) roller cracked bearings.

Take the first component of different types of signals decomposed by VMD as an example. The fitness curves of the WGWOA, WOA, and GWO algorithms in VMD optimization are compared, and the number of iterations of the algorithm is 50 to verify the feasibility of WGWOA algorithm in optimizing the VMD parameters. As shown in Figure 13, three different algorithms are used to optimize the fitness curves of VMD.

From Figure 13, the WOA algorithm has the highest adaptability in the bearing signal decomposition of four fault types, and the solution may be local optimum, which proves that the WOA algorithm is easy to fall into local optimum. The adaptability of GWO algorithm is lower than that of the WOA algorithm, which indicates that GWO has a stronger global optimization ability than WOA, but it converges only when the number of iterations is higher, and its convergence ability is lower than that of WOA. However, the adaptability of the WGWOA algorithm converges to a lower value when the number of iterations is low. This condition is because the WGWOA algorithm introduces the position updating method of WOA algorithm based on the GWOA algorithm, which has the convergence performance and the ability of global optimization. This finding proves the feasibility of the WGWOA algorithm to optimize VMD.

Therefore, the WGWOA algorithm mentioned in this paper is used to optimize the parameters *K* and *σ* of VMD and label various fault types to facilitate later training of the fault diagnosis model. The optimal parameter *K* and *σ* solutions for different fault types were obtained, as shown in Table 5.

**Figure 14:**
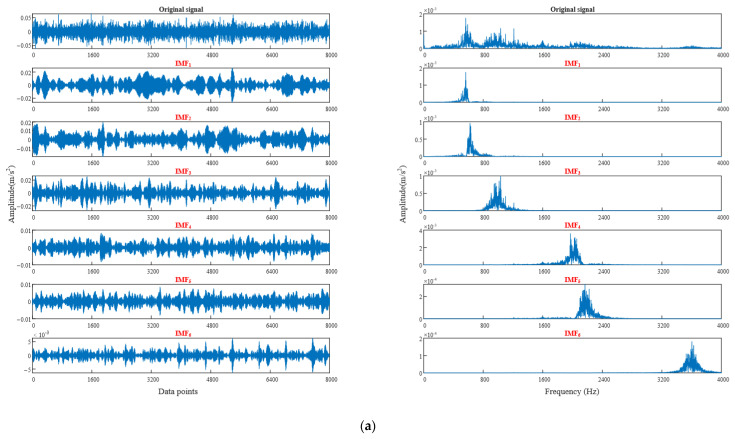

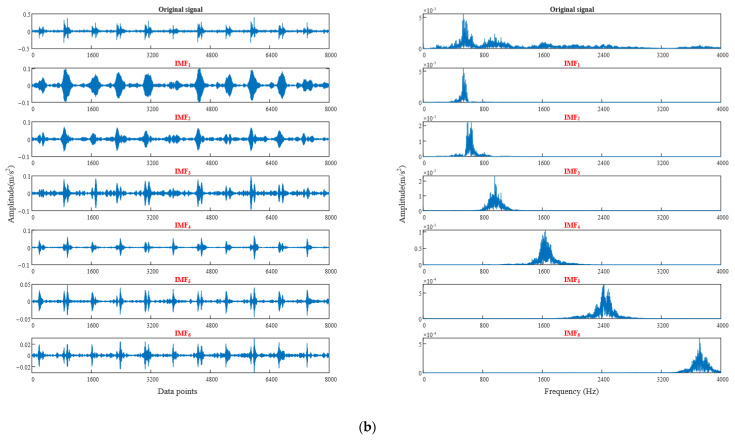
Optimizing VMD to decompose the time-domain waveform and spectrum of vibration signals of different fault types, (**a**) normal, (**b**) inner ring crack.

From Table 5, the optimum decomposition levels *K* of VMD for four fault type signals are all 6, optimized *σ* values are all around 4800. The method in Section 4.2 is adopted, the average value of *σ* is 4829.25 to form the optimal parameter combination [4829.25, 6]. The rolling bearing data of different fault types are decomposed through VMD by using the optimal parameter combination. The time-domain waveform and frequency spectrum of vibration signal after optimized VMD are shown in Figure 14. Only the vibration signal decomposition of the normal bearing and the bearing with damaged inner ring is listed here due to the length of the article.

As shown in Figure 14, the IMF components of the two fault signals after VMD do not produce modal aliasing, which further verifies the feasibility of the WGWOA algorithm to optimize VMD.

#### 5.2.3. Feature Extraction

In accordance with the proposed method of extracting eigenvalues, this paper extracts the permutation entropy of each component decomposed by VMD as the eigenvector *F_v_*, so as to construct the data of SVM training samples and test samples. The expression of *F_v_* is as follows:

(25) $$F_{v}={\left[PE\left({\mathrm{IMF}}_{1}\right),PE\left({\mathrm{IMF}}_{2}\right),PE\left({\mathrm{IMF}}_{3}\right),PE\left({\mathrm{IMF}}_{4}\right),PE\left({\mathrm{IMF}}_{5}\right),PE\left({\mathrm{IMF}}_{6}\right)\right]}^{T}$$

The resulting data set is shown in Table 6.

Although a certain correlation is found between the permutation entropy of each component and the fault type, the specific relationship between them is more complex, and visually observing what fault type the eigenvector represents is impossible.

### 5.3. Fault Diagnosis Based on WGWOA-Optimized SVM

**Figure 15:**
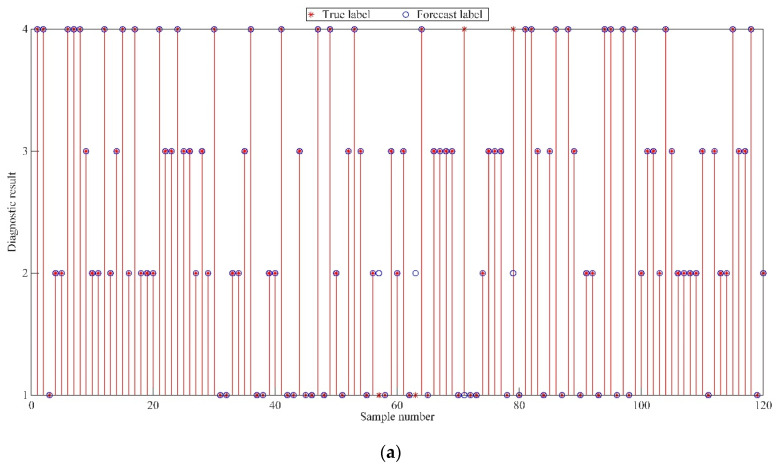

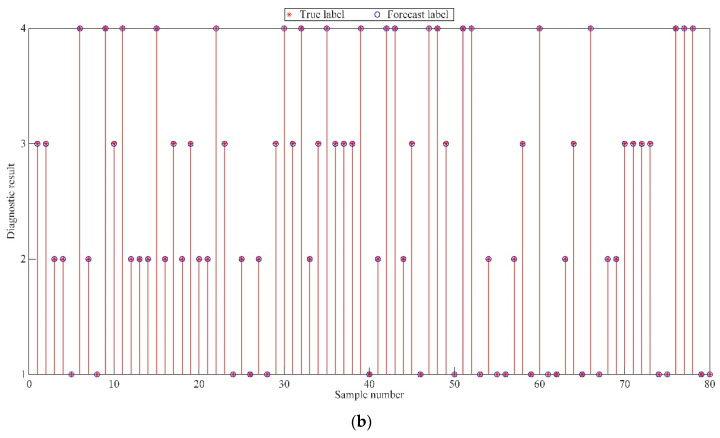
Diagnostic results of different samples, (**a**) training samples, (**b**) test samples.

This paper uses SVM as the fault diagnosis model due to its powerful ability to process complex data. The proposed WGWOA algorithm is utilized to optimize its parameters *c* and *g*. The vibration signal eigenvector is processed in accordance with the method in Section 5.2.1 to construct the training and test samples, so as to train the SVM. The classification results of training samples and the diagnostic results of test samples are shown in Figure 15 and Table 7.

As shown in Figure 15 and Table 7, only four sample points failed to be correctly classified in the SVM training process, the diagnostic accuracy of the training samples reached 96.67%, and the classification accuracy of the test samples reached 100%. Combined with the diagnostic accuracy of the two samples, the optimized SVM did not exhibit fitting phenomenon. The proposed optimization method of the permutation entropy characteristic matrix of each mode after VMD is scientific and effective because VMD can effectively avoid the phenomenon of signal mode aliasing, and the decomposed multiple modes are distinguished. The WGWOA algorithm is used to find the best *K* and *σ* parameter combination, which enhances the availability of VMD to extract the permutation entropy feature vector. The reliability of applying the WGWOA algorithm to the optimization of SVM parameters *c* and *g* is verified. This finding is because the WGWOA algorithm can efficiently and accurately find the optimal parameters *c* and *g* of SVM and build a high-performance SVM model to avoid over fitting and over learning.

### 5.4. Comparative Analysis with Other Methods

**Figure 16:**
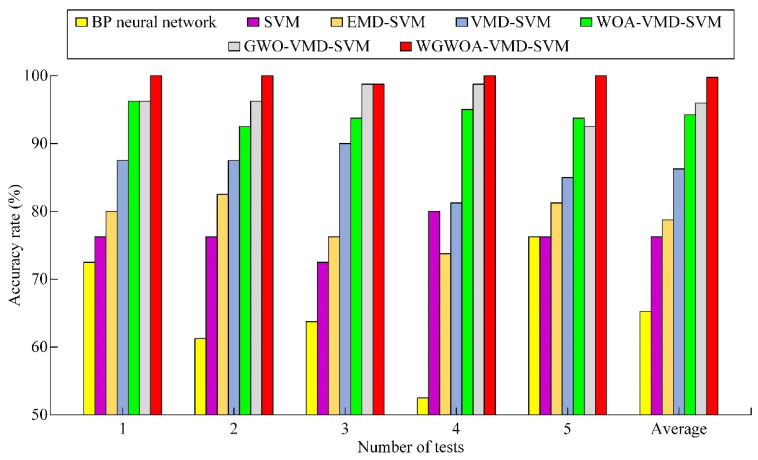
Test diagnostic accuracy.

The proposed fault diagnosis method was compared with the fault diagnosis methods of BPNN, SVM, EMD-SVM, VMD-SVM, WOA simultaneously optimizing VMD, SVM model (WOA-VMD-SVM), GWO simultaneously optimizing VMD, and SVM model (GWO-VMD-SVM) to verify its effectiveness and practicability. The above seven fault diagnosis methods were used for five experiments to increase the reliability of the experimental results and to avoid their randomness. The diagnostic results are shown in Table 8 and Figure 16.

**Figure 17:**
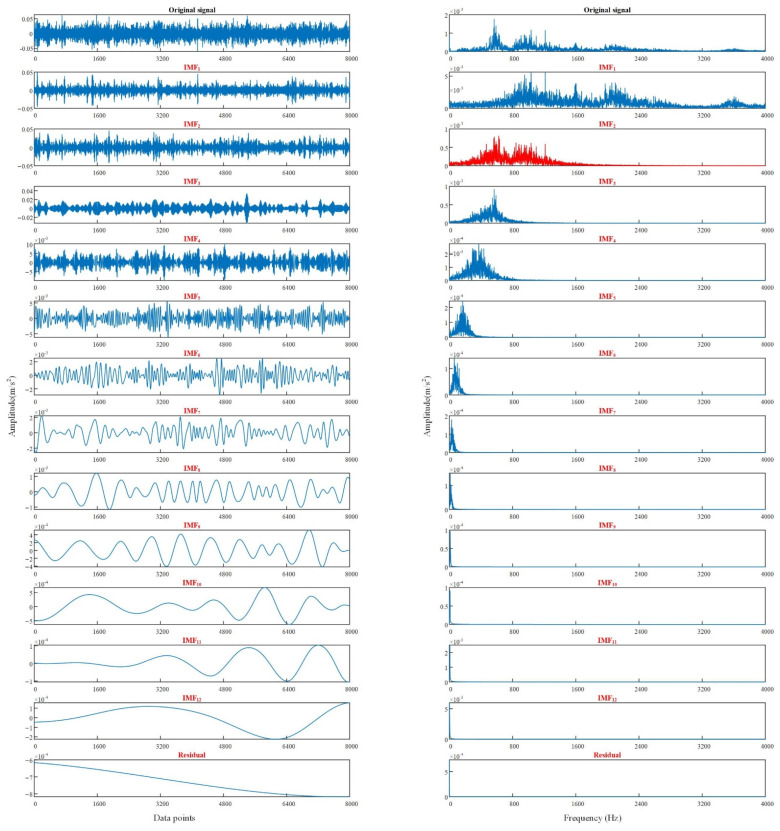
EMD result of normal bearing vibration signal.

As shown in Table 8 and Figure 16, the convergence speed of BPNN is slow, and the network performance is biased compared with SVM. The generalization ability of the network is poor under the small sample data, resulting in a draw accuracy of only 65.25%. This finding proves that the SVM fault diagnosis model has strong robustness under the small sample data. The average fault diagnosis rate of EMD-SVM model is 78.75%, which is higher than that of the SVM model. However, taking the normal bearing as an example, the peaks in the IMF component of its vibration signal decomposed by EMD appear at about 500 Hz and 1000 Hz in the IMF_2_ spectrum, and modal aliasing occurs. EMD decomposes 12 IMF components (the last one is residual), and some component signals are arranged disorderly. This finding shows that decomposing noncharacteristic false components is extremely possible and extracting eigenvalues from the decomposed false components certainly increases the recognition difficulty of the fault diagnosis model. The average fault diagnosis rate of the VMD-SVM model reaches 86.25%, which is 10% higher than that of SVM. Combined with Figure 14 and Figure 17, VMD has superior performance, and EMD is more suitable for actual fault diagnosis.

**Figure 18:**
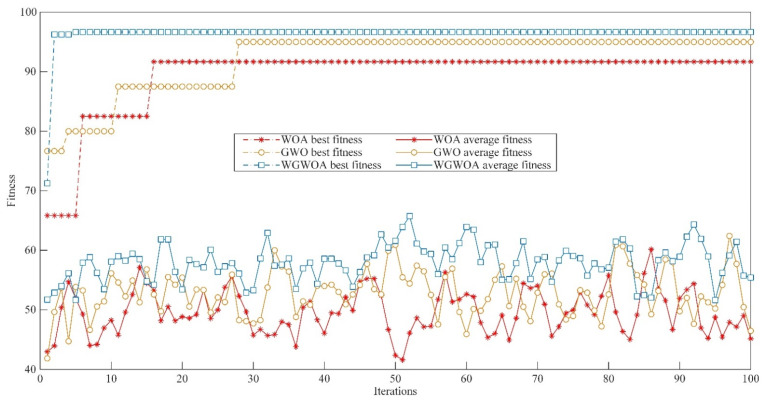
Different algorithms used to optimize the fitness curve of SVM.

Although the VMD-SVM model is better than the BPNN, SVM, and EMD-SVM, it does not scientifically select the parameters of VMD and SVM, resulting in a fault diagnosis rate of less than 90.00%, and the model performance still needs to be improved. The average fault diagnosis of the model reaches 94.25% after optimizing the parameters of VMD and SVM with the WOA algorithm. This finding indicates that the WOA algorithm can play a certain role in the parameter optimization of VMD and SVM. However, the best *c* and *g* solutions obtained by WOA are 4.23 and 0.01 by observing Figure 18 and Table 9. Taking the cross-validation during the training of SVM as the fitness, the best and average fitness curves of WOA algorithm are maintained at a low level, and the best fitness convergence value is 91.67. For the other two algorithms, the SVM parameters obtained by the WOA algorithm may be local optimum. The best *c* and *g* solutions found by GWO are 15.32 and 0.22. Compared with WOA, the GWO algorithm has the best and higher average fitness curve, but it converges 28 times during iteration, which shows that the convergence of GWO algorithm is slower than that of the WOA algorithm, which is the same as that in Section 5.2.2. The WGWOA algorithm only converges to 96.67 at the best fitness of five generations. Compared with the WOA and GWO algorithms, the best and average fitness of the WGWOA algorithm are maintained at a high level. The average diagnostic rate of the WGWOA-VMD-SVM model for five repeated tests is 99.75%, which verifies the superiority of the WGWOA algorithm in SVM optimization. In conclusion, the proposed WGWOA-VMD-SVM method has many advantages, such as high efficiency and high accuracy, to meet the practical application requirements.

## 6. Conclusions

In this paper, a fault diagnosis method combining VMD and SVM is adopted, and a WGWOA algorithm is proposed to optimize the *K* and *σ* parameters of VMD and the *c* and *g* parameters of SVM. The permutation entropy feature matrix is extracted, and the SVM is trained and verified by collecting the vibration signals of rolling bearings with different fault types for preprocessing. The conclusions after comparing the proposed method with several existing fault diagnosis methods are as follows:

1. The test results of two cases show that WGWOA-optimized VMD can properly suppress modal aliasing and that WGWOA-optimized SVM enhances the accuracy and self-adaptability of model classification. The average accuracy of this method in five repeated tests were 100.00% and 99.75%. Compared with other existing fault diagnosis methods, this method has many advantages, such as high accuracy and stable performance, to provide an effective new method for the existing fault diagnosis technology;
2. Compared with other optimization algorithms, the proposed WGWOA algorithm has good performance in terms of optimization accuracy, optimization efficiency, and algorithm convergence. The training process of this method is simple and fast, and the diagnostic accuracy after training is significantly higher than other traditional methods.

## Figures and Tables

**Table 1:** Sample Settings.

Fault Types	Load/(hp)	Number of Training Samples	Number of Test Samples	Sample Number
Normal	0	45	15	1
	1			
	2			
Inner ring fault (fault diameter 0.1778 mm)	0	45	15	2
	1			
	2			
Inner ring fault (fault diameter 0.3556 mm)	0	45	15	3
	1			
	2			
Inner ring fault (fault diameter 0.5334 mm)	0	45	15	4
	1			
	2			
Outer ring fault (fault diameter 0.1778 mm)	0	45	15	5
	1			
	2			
Outer ring fault (fault diameter 0.3556 mm)	0	45	15	6
	1			
	2			
Outer ring fault (fault diameter 0.5334 mm)	0	45	15	7
	1			
	2			
Rolling element fault (fault diameter 0.1778 mm)	0	45	15	8
	1			
	2			
Rolling element fault (fault diameter 0.3556 mm)	0	45	15	9
	1			
	2			
Rolling element fault (fault diameter 0.5334 mm)	0	45	15	10
	1			
	2			

**Table 2:** Optimal parameter solutions.

Fault Types	Optimum Solutions	
	*σ*	*K*
Normal	2012	4
Inner ring fault (fault diameter 0.1778 mm)	1999	4
Inner ring fault (fault diameter 0.3556 mm)	1982	4
Inner ring fault (fault diameter 0.5334 mm)	2003	4
Outer ring fault (fault diameter 0.1778 mm)	1996	4
Outer ring fault (fault diameter 0.3556 mm)	1988	4
Outer ring fault (fault diameter 0.5334 mm)	1999	4
Rolling element fault (fault diameter 0.1778 mm)	2007	4
Rolling element fault (fault diameter 0.3556 mm)	1987	4
Rolling element fault (fault diameter 0.5334 mm)	1989	4
Optimum parameter combination	1996.20	4

**Table 3:** Diagnostic accuracy of different methods.

Methods	Accuracy (%)					
	Experiment 1	Experiment 2	Experiment 3	Experiment 4	Experiment 5	Average
VMD-SVM	97.33	96.00	98.66	94.00	97.33	96.66
WGWOA-VMD-SVM	100.00	100.00	100.00	100.00	100.00	100.00

**Table 4:** Specifications and parameters of test bearings.

Types	Specifications	Outer Diameter/mm	Inside Diameter/mm	Thickness/mm	Rollers Number	Roller Diameter/mm	Pitch/mm	Contact Angle/°
Cylindrical roller bearing	N205EM	52	25	15	13	6.5	38.5	0

**Table 5:** Optimal parameter *K* and *σ* solutions for different fault types.

Fault Types	Optimum Solutions		Labels
	*σ*	*K*	
Normal	4835	6	1
Inner ring crack	4862	6	2
Outer ring crack	4822	6	3
Roller crack	4798	6	4

**Table 6:** Permutation entropy eigenvalue of vibration signal extraction.

Fault Types	Permutation Entropy					
	IMF_1_	IMF_2_	IMF_3_	IMF_4_	IMF_5_	IMF_6_
Normal	1.5309	1.0415	1.5753	0.8844	1.3019	0.1141
	1.3952	1.1453	1.7707	1.2923	1.3228	0.1434
	1.4194	1.0538	1.8166	0.9955	1.1806	0.1031
	1.2777	1.0539	1.8729	0.9517	1.0573	0.1127
	1.3725	1.1848	1.9700	1.0411	1.3821	0.1062
Inner ring crack	1.4377	1.6552	1.5502	0.8704	0.8514	0.1254
	1.4575	1.3057	2.0078	0.7103	0.8670	0.1349
	1.3202	1.5059	1.8016	0.8518	1.0440	0.1048
	1.2304	1.4806	1.8627	0.9084	1.0683	0.1600
	1.4751	1.4448	1.5748	0.7900	0.8558	0.1218
Outer ring crack	2.4565	2.2353	2.2428	1.5334	2.6846	0.3680
	1.7272	2.5163	2.0670	1.7688	2.7149	0.3893
	1.7458	2.4905	2.2135	1.5629	2.6006	0.4480
	1.7611	2.4239	2.1152	1.4379	2.5880	0.4388
	1.7432	2.4397	2.2388	1.5594	2.6276	0.4215
Roller crack	0.8964	1.4693	1.9504	1.3214	1.2628	0.2148
	1.2302	1.1797	2.1101	0.9923	1.7222	0.3233
	1.1171	1.3704	1.9537	0.8106	1.4210	0.2868
	1.2216	0.8641	2.0169	0.9422	1.4858	0.2865
	1.3158	1.1262	1.8119	0.9066	1.5658	0.2998

**Table 7:** Diagnostic error types of different samples.

Sample Types	Sample Point Label of Diagnostic Error	Actual Fault Types	Diagnostic Fault Types	Diagnostic Accuracy
Training sample	57	Normal	Inner ring crack	96.67%
	63	Normal	Inner ring crack	
	71	Roller crack	Normal	
	79	Roller crack	Inner ring crack	
Test sample	-	-	-	100.00%

**Table 8:** Diagnostic accuracy of different method tests.

Methods	Accuracy (%)					
	Experiment 1	Experiment 2	Experiment 3	Experiment 4	Experiment 5	Average
BPNN	72.50	61.25	63.75	52.50	76.25	65.25
SVM	76.25	76.25	72.50	80.00	76.25	76.25
EMD-SVM	80.00	82.50	76.25	73.75	81.25	78.75
VMD-SVM	87.50	87.50	90.00	81.25	85.00	86.25
WOA-VMD-SVM	96.25	92.50	93.75	95.00	93.75	94.25
GWO-VMD-SVM	96.25	96.25	98.75	98.75	92.50	96.50
WGWOA-VMD-SVM	100.00	100.00	98.75	100.00	100.00	99.75

**Table 9:** Optimal solution of SVM parameters found by different algorithms.

Optimization Algorithms	Optimal Solutions	
	*c*	*g*
WOA	4.23	0.01
GWO	15.32	0.22
WGWOA	25.78	2.48

## Data Availability

Not applicable.
